# SARS-CoV-2 vaccination-infection pattern imprints and diversifies T cell differentiation and neutralizing response against Omicron subvariants

**DOI:** 10.1038/s41421-022-00501-3

**Published:** 2022-12-21

**Authors:** Junxiang Wang, Kaiyi Li, Xinyue Mei, Jinpeng Cao, Jiaying Zhong, Peiyu Huang, Qi Luo, Guichang Li, Rui Wei, Nanshan Zhong, Zhuxiang Zhao, Zhongfang Wang

**Affiliations:** 1 State Key Laboratory of Respiratory Disease & National Clinical Research Center for Respiratory Disease, Guangzhou Institute of Respiratory Health, the First Affiliated Hospital of Guangzhou Medical University, Guangzhou Medical University, Guangzhou, Guangdong China; 2 Guangzhou Laboratory, Bioland, Guangzhou, Guangdong China; 3 Department of Infectious Disease, Respiratory and Critical Care Medicine, Guangzhou First People’s Hospital, Guangzhou Medical University, Guangzhou, Guangdong China

**Keywords:** Immunology, Mechanisms of disease

## Abstract

The effects of different SARS-CoV-2 vaccinations and variant infection histories on imprinting population immunity and their influence on emerging escape mutants remain unclear. We found that Omicron (BA.1) breakthrough infection, regardless of vaccination with two-dose mRNA vaccines (M-M-o) or two-dose inactivated vaccines (I-I-o), led to higher neutralizing antibody levels against different variants and stronger T-cell responses than Delta breakthrough infection after two-dose inactivated vaccine vaccination (I-I-δ). Furthermore, different vaccination-infection patterns imprinted virus-specific T-cell differentiation; M-M-ο showed higher S/M/N/E-specific CD4^+^ T cells and less portion of virus-specific CD45RA^+^CD27^–^CD8^+^ T cells by ex vivo assay. Breakthrough infection groups showed higher proliferation and multi-function capacity by in vitro assay than three-dose inactivated vaccine inoculated group (I-I-I). Thus, under wide vaccination coverage, the higher immunogenicity with the Omicron variant may have helped to eliminate the population of Delta variant. Overall, our data contribute to our understanding of immune imprinting in different sub-populations and may guide future vaccination programs.

## Introduction

Omicron variant infection was reported for the first time on November 26, 2021^1^. By March 31, 2022, the Omicron variant had been detected in 188 countries and had already become the globally dominant variant, accounting for 99.7% of submitted sequences from February 23 to March 24, 2022^2^. Thus, the Omicron variant almost completely replaced Delta variant and became the dominant circulating variant worldwide^3^. This occurred soon after the worldwide use of COVID-19 vaccines^4^. Retrospective studies have confirmed an increased breakthrough infection rate for that SARS-CoV-2, with thus lower vaccine efficacy over time via evolution from the prototype Delta variant to the Omicron variant^5–7^. For example, based on data received through June 27, 2022, 1,620,244 laboratory-confirmed breakthrough cases of COVID-19 among fully vaccinated people were reported in New York State, where mRNA vaccines were prevalently used, corresponding to 12.1% of the fully vaccinated population aged 12 years or older^8^. For different subgroups based on variant and time point, the vaccine exhibited a protective efficacy of 92.8% for the prototype strain as of May 3, 2021, 80% after the Delta variant became predominant in mid-July 2021, and 68.7% for the Omicron variant since December 13, 2021^9^. The 21 mutations in the receptor-binding domain (RBD)^6^, which provided exceptional immune escape capacity against vaccine-induced immunity, combined with the high transmissibility index R0 of 9.1^10,11^ explained why the Omicron variant could spread quickly among vaccinated people. However, the reason why the Delta variant cannot coexist (cocirculate) with the Omicron variant remains unexplained. Therefore, it is interesting to study whether Omicron variant-induced specific host immunity under widespread vaccine use was involved in the rapid worldwide replacement of the circulating Delta variant in such a short period.

Studying the difference in host immune response under different patterns (infection only, vaccination only, vaccination after infection, and infection after different vaccination strategies) can provide a good perspective for understanding dynamic interactions between emerging variants, vaccinations, and the extent/quality/level of sub-population immunity. Studies have shown that SARS-CoV-2 natural infection-induced antibodies continue to grow in potency as well as breadth against variants for one year after infection; in contrast, most antibodies elicited by vaccination appear to stop changing in weeks after the second dose^12,13^. Memory B cells that evolve after infection were also more likely to produce antibodies that block immune-evading variants such as Beta and Delta variants than those developed after vaccination^14–16^. Compared to people with vaccination only or infection only, patients who recovered from COVID-19 with later vaccination were reported to produce higher levels of anti-spike IgG antibodies and neutralizing antibodies (NAbs) with broader neutralizing capacity against different SARS-CoV-2 variants of concern (VOCs)^17,18^. Further analysis has revealed more mutations in long-lived memory B cells from patients who have recovered, which continued to evolve by affinity maturation in lymph nodes over time; after re-exposure to mRNA vaccination, these high-affinity memory B cells multiplied rapidly and produced more highly potent antibodies^12,19^.

Inactivated vaccines are crucial against the Alpha, Beta, and Delta variants^20^ and are widely used. Compared to mRNA, subunit and adenovirus vector vaccines, inactivated whole-virus vaccines contain viral protein antigens other than the S protein or the RBD alone and were reported to induce lower levels of NAbs against the Omicron variant after two prime doses and a booster dose^21,22^. This situation indicated a higher possibility of Omicron variant breakthrough infection after inactivated vaccine vaccination with more severe and fatal cases. Surprisingly, recent studies have shown that three-dose inactivated vaccine could provide protection efficacy of 98.1% against Omicron-induced severe symptoms and death, similar to the 98.3% efficacy of the mRNA vaccine, in elderly people (> 60 years old)^23–25^. The inactivated vaccine was also found to have better protective efficacy in the real world than expected based on an immunogenicity study. Because Omicron subvariants such as BA.1, BA.2, BA.4/5 have gradually acquired greater immunity escape capacity, Omicron breakthrough infection will likely eventually occur in those vaccinated with inactivated vaccines. Therefore, it is of great interest to investigate the level of sub-population immunity within sub-populations with Omicron breakthrough infection after receiving two-dose inactivated vaccine.

In this study, we compared the immune response to breakthrough infections by two variants, Delta and Omicron, after two-dose inactivated vaccine with respect to levels of NAbs and T-cell immune responses against wild-type (WT) strain-specific peptide pools. We also examined T-cell responses in different vaccination-infection patterns. Overall, our results raised a possibility why the Delta variant did not cocirculate with the Omicron variant from the perspective of the host immune response. These results may guide future studies to find a safer path to sub-population immunity with a higher level of protection.

## Results

### NAb levels upon Omicron breakthrough infection exceeded those upon Delta breakthrough infection in vaccinated individuals

**Fig. 1:**
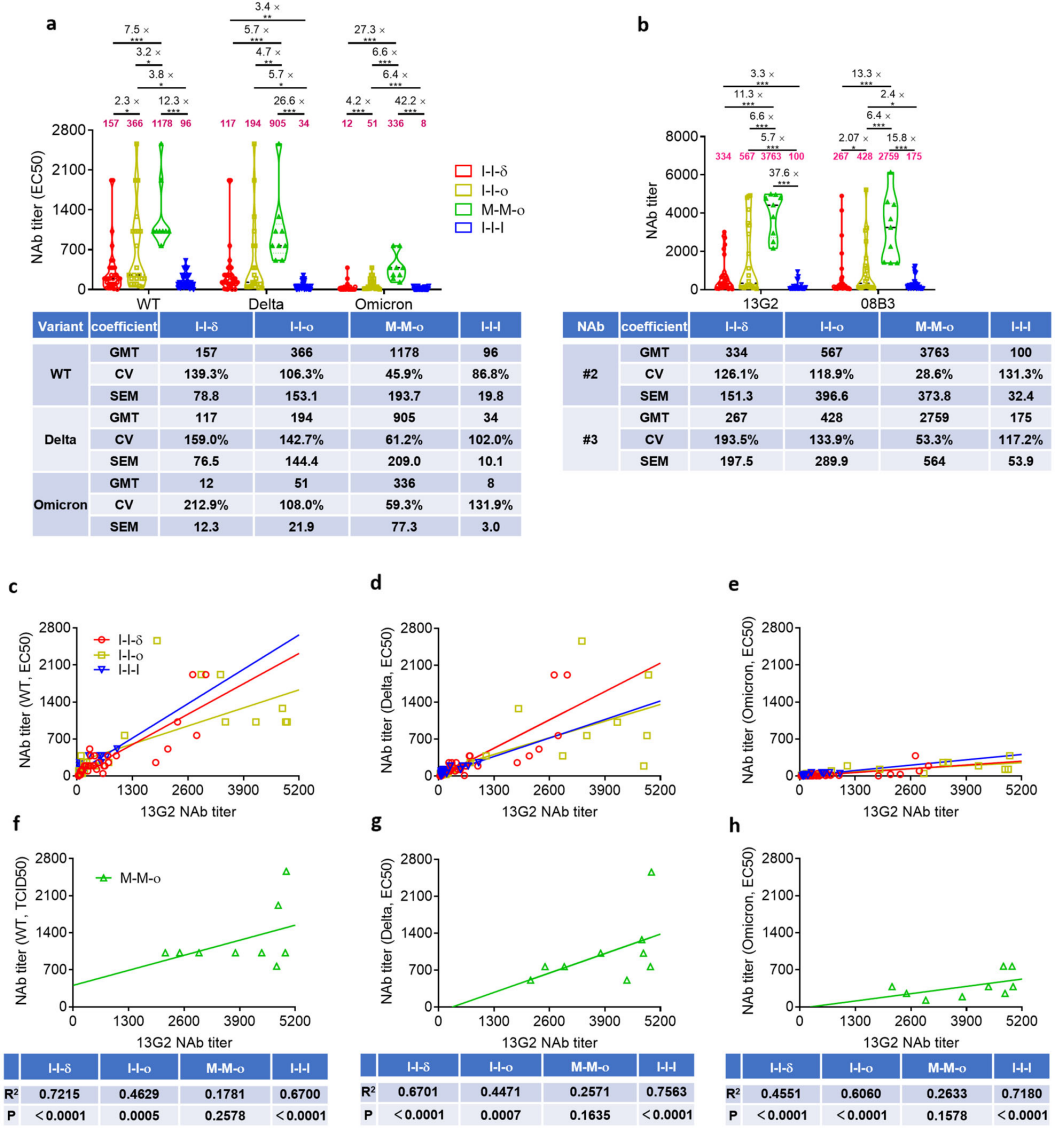
Comparison of neutralizing activity among I-I-δ, I-I-ο, M-M-ο, and I-I-I groups. **a** Neutralizing response against WT SARS-CoV-2 and VOCs, including Delta and Omicron variants. The numbers in magenta indicated the geometric mean titers (GMTs), and fold change in GMT for the virus compared with the strain with lower neutralizing titer. **b** Comparison of NAb titers based on the monoclonal antibodies 13G2 and 08B3 among I-I-δ, I-I-ο, M-M-ο, and I-I-I groups. **c**–**h** Correlation of NAbs against the WT and the Delta and Omicron variants and levels of the monoclonal antibody 13G2 among I-I-δ, I-I-ο, M-M-ο and I-I-I groups. Significance was measured using the Mann–Whitney tests. *P < 0.05, **P < 0.01, ***P < 0.001. I-I-δ, Delta infection after two-dose inactivated vaccine (*n* = 34); I-I-o, Omicron infection after two-dose inactivated vaccine (*n* = 22); M-M-o, Omicron infection after two-dose mRNA vaccination (*n* = 9); I-I-I, three-dose inactivated vaccine (*n* = 40).

To investigate whether host immunity was involved in the rapid replacement globally of circulating Delta variant in a short period, we measured NAb levels against different variants in plasma samples of individuals with Delta infection after two-dose inactivated vaccine (I-I-δ, *n* = 34), Omicron infection after two-dose inactivated vaccine (I-I-ο, *n* = 22), Omicron infection after two-dose mRNA vaccine (M-M-ο, *n* = 9), and after three-dose inactivated vaccine (I-I-I, *n* = 40). Our results demonstrated much higher levels of cross-NAbs induced by I-I-ο than by I-I-δ against WT (366 vs 157), Delta (194 vs 117), and Omicron (51 vs 12). Interestingly, Delta breakthrough infection induced notably lower levels of NAbs (12) against the Omicron variant. In contrast, Omicron breakthrough infection induced rather higher NAb levels against Delta variant (194). Our data suggested that the Omicron variant could potentially build the stronger immune barrier with higher NAb levels to combat Delta re-infection, likely to be one of the multiple factors, such as viral stability, transmissibility, immune-evasive capability, immunological properties, survival selective pressure and immunity pressure, to work in concert to determine the fate of Delta variant replacement by Omicron variant. Surprisingly, M-M-ο induced a 7.5-fold, 5.7-fold, and 27.3-fold increase in NAb levels compared with I-I-δ against the WT (1178 vs 157, *P* < 0.0001), Delta (905 vs 117, *P* < 0.0001), and Omicron (336 vs 12, *P* < 0.0001) variants. This revealed that M-M-ο could provide much better protection against I-I-δ but that I-I-δ could not prevent re-infection by Omicron variant. Similarly, M-M-ο induced much higher levels of NAbs against WT (3.2-fold, 1178 vs 366, *P* = 0.0223), Delta (4.7-fold, 905 vs 194, *P* = 0.0072), and Omicron (6.6-fold, 336 vs 51, *P* < 0.0001) than I-I-ο (Fig. 1a). Therefore, mRNA-vaccinated individuals could establish better sub-population immunity after Omicron breakthrough infection than those immunized with inactivated vaccine. This further strengthened the hypothesis that Omicron breakthrough infection could establish stronger sub-population immunity reflected by higher level of NAbs than Delta breakthrough infection even after different vaccination strategies, thus giving a possible explanation that the Omicron variant replaced the Delta variant under the circumstance of different widely used vaccines from host immune response.

To investigate the components and breadth of NAbs induced by different breakthrough infections in an individual, we measured NAb levels represented by two individual monoclonal antibodies. Antibody 13G2 and antibody 08B3, which target two different B-cell epitopes on the S protein RBD of the WT strain, were the two potent NAbs that emerged during our early screening process and were detectable in most of the vaccinated population and SARS-CoV-2-infected patients. Our previous data showed that two NAb clusters represented by 13G2 and 08B3 were selected and became present at higher levels at 12 months than at 2 months after prototype infection. In the present study, our data showed that M-M-ο induced 11.3-fold and 13.3-fold higher levels of NAbs 13G2 and 08B3 than I-I-δ; I-I-ο induced 2.07-fold higher 08B3 level than I-I-δ (Fig. 1b). Moreover, M-M-ο induced higher levels of 13G2 (6.6-fold, 37.6-fold) and 08B3 (6.4-fold, 15.8-fold) than I-I-ο and I-I-I, respectively, showing that Omicron infection after mRNA vaccination resulted in much better protective immunity. The data for individual NAb levels included observations at the level of total NAbs. All the above data indicated that Omicron breakthrough infection led to higher levels of protective antibodies than Delta infection and confirmed that mRNA vaccination induced higher sub-population immunity, even after breakthrough infection.

We also sought to identify any correlation between titers of total NAbs and 13G2 or 08B3 for WT, Delta, and Omicron variants in the four groups. Correlation analysis revealed that the titer of total NAbs correlated with that of 13G2 or 08B3 in I-I-δ, I-I-ο, and I-I-I, even though the former two groups showed a decreasing trend of correlation for WT, Delta, and Omicron variants and the latter one group did not. Surprisingly, only the titers of WT and Delta NAbs correlated with 08B3 NAb titer in M-M-o group (Fig. 1c–h; Supplementary Fig. S1). Our findings suggested that 13G2 and 08B3 predominated over inactivated vaccine-induced NAbs and that an inactivated vaccine still played a dominating role in immune imprinting despite some changes in the antibody spectrum after breakthrough infection by different variants. Moreover, the fact that no correlation of 13G2 and NAb titers was found in M-M-ο group indicated an entirely different NAb spectrum for the mRNA vaccine compared to the inactivated vaccine. In general, the NAb titers further suggested that the mRNA vaccine induced a broader spectrum of NAbs.

### M-M-ο induced the highest level of NAbs against BA.4/5 based on pseudovirus neutralization assay

**Fig. 2:**
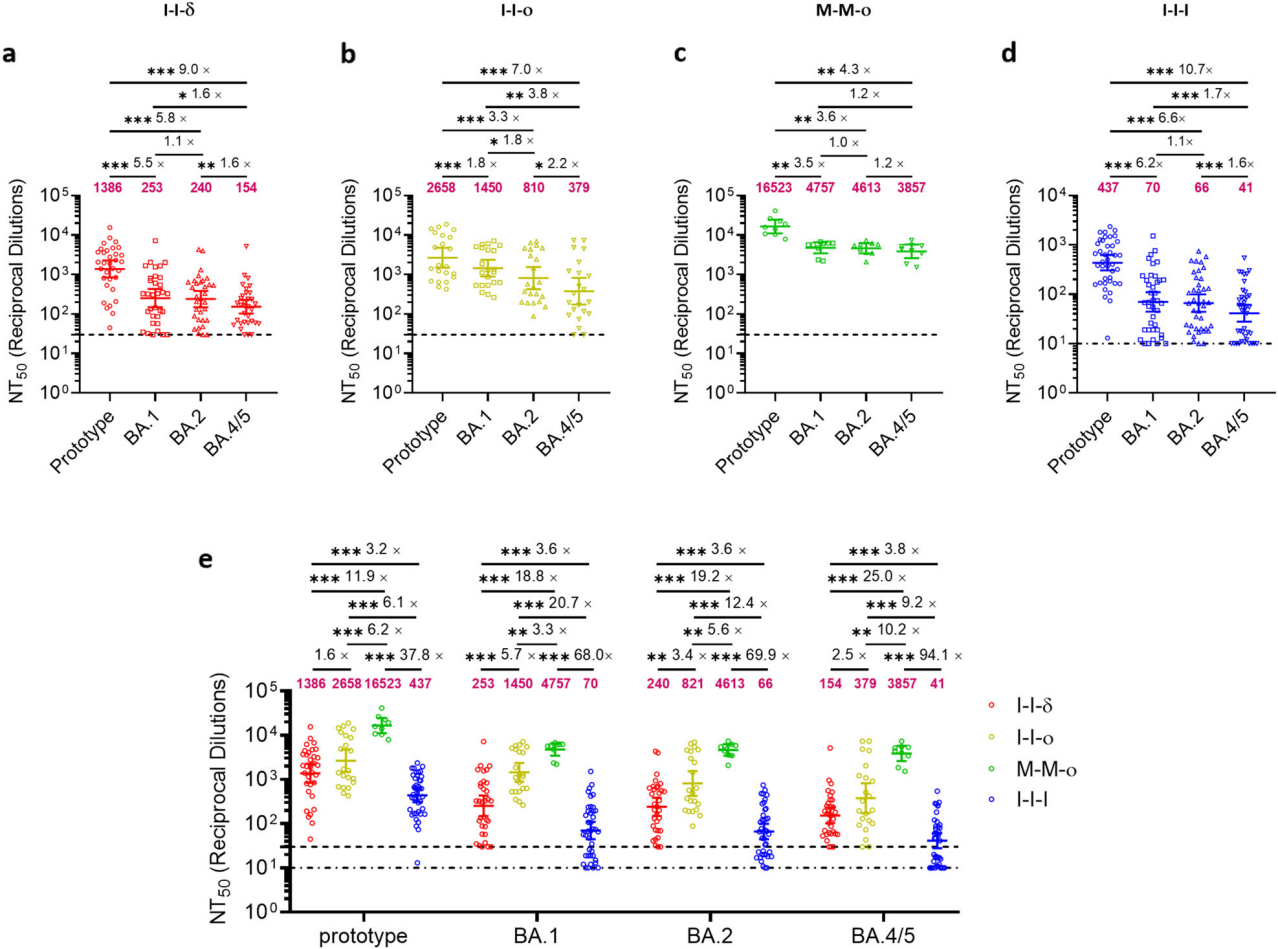
The pseudovirus NAb titer against prototype, BA.1, BA.2 and BA.4/5. **a**–**d** Plasma from I-I-δ (*n* = 33), I-I-o (*n* = 22), M-M-o (*n* = 9) and I-I-I (*n* = 40) groups were assessed for pseudovirus NAb titers. **e** Comparison of NAb titers against prototype, BA.1, BA.2 and BA.4/5 among 4 groups. Geometric mean NT_50_ values and fold change were displayed at the top of plots; bars represented geometric mean ± 95% confidence interval (CI), and significance relative to each Pseudovirus was determined by Wilcoxon rank-sum test. *P < 0.05, **P < 0.01, ***P < 0.001. Dash line in **a**–**c** means that the limit of detection (LOD) of NT_50_ in I-I-δ, I-I-o, M-M-o groups was 30; dash-dotted line in **d** means that the LOD of NT_50_ in I-I-I group was 10.

To investigate whether the immunity induced by different vaccination-infection patterns was still effective against new emerging Omicron variants, especially recent pandemic candidate BA.4/5, based on pseudovirus neutralization assay, we measured the NAb levels against prototype and different variants (BA.1, BA.2, BA.4/5) for I-I-δ (*n* = 33), I-I-o (*n* = 22), M-M-o (*n* = 9) and I-I-I (*n* = 40). Comparing the NAb levels between prototype and BA.4/5, four groups decreased by 9.0-fold (I-I-δ, 1386 vs 154), 7.0-fold (I-I-o, 2658 vs 379), 4.3-fold (M-M-o, 16523 vs 3857) and 10.7-fold (I-I-I, 437 vs 41) (Fig. 2a–d). Among the 4 groups, M-M-o induced the highest NAb level (3857) against BA.4/5, which was 25.0-fold, 10.2-fold, and 94.1-fold higher than I-I-δ, I-I-o, and I-I-I, respectively, followed by I-I-o (379, 2.5-fold higher than I-I-δ and 9.2-fold higher than I-I-I), and I-I-δ (154, 3.8-fold higher than I-I-I) (Fig. 2e). Omicron breakthrough infection, either after two-dose inactivated vaccines or two-dose mRNA vaccines, induced higher NAb titers against BA.4/5 than Delta breakthrough infection. On the other hand, although BA.4/5 derived from BA.2, we could see that NAb level against BA.2 was significantly higher than BA.4/5 among I-I-δ (1.6-fold, *P* = 0.0050), I-I-o (2.2-fold, *P* = 0.0024), M-M-o (1.2-fold, *P* = 0.4258) and I-I-I (1.6-fold, *P* = 0.0003) (Fig. 2a–d), indicating an immune escape from BA.2 to BA.4/5. Therefore, our results partially explained why BA.4/5 could quickly become dominant after BA.2 spread. In a word, our results showed that although the NAb levels declined from prototype to BA.4/5, M-M-o could provide a much higher NAb level against Omicron sub-lineages compared to I-I-o or I-I-δ, which suggested Omicron breakthrough infection and mRNA vaccine could provide better immunogenicity.

### M-M-ο induced stronger virus-specific CD4^+^ and CD8^+^ T-cell responses than I-I-I

**Fig. 3:**
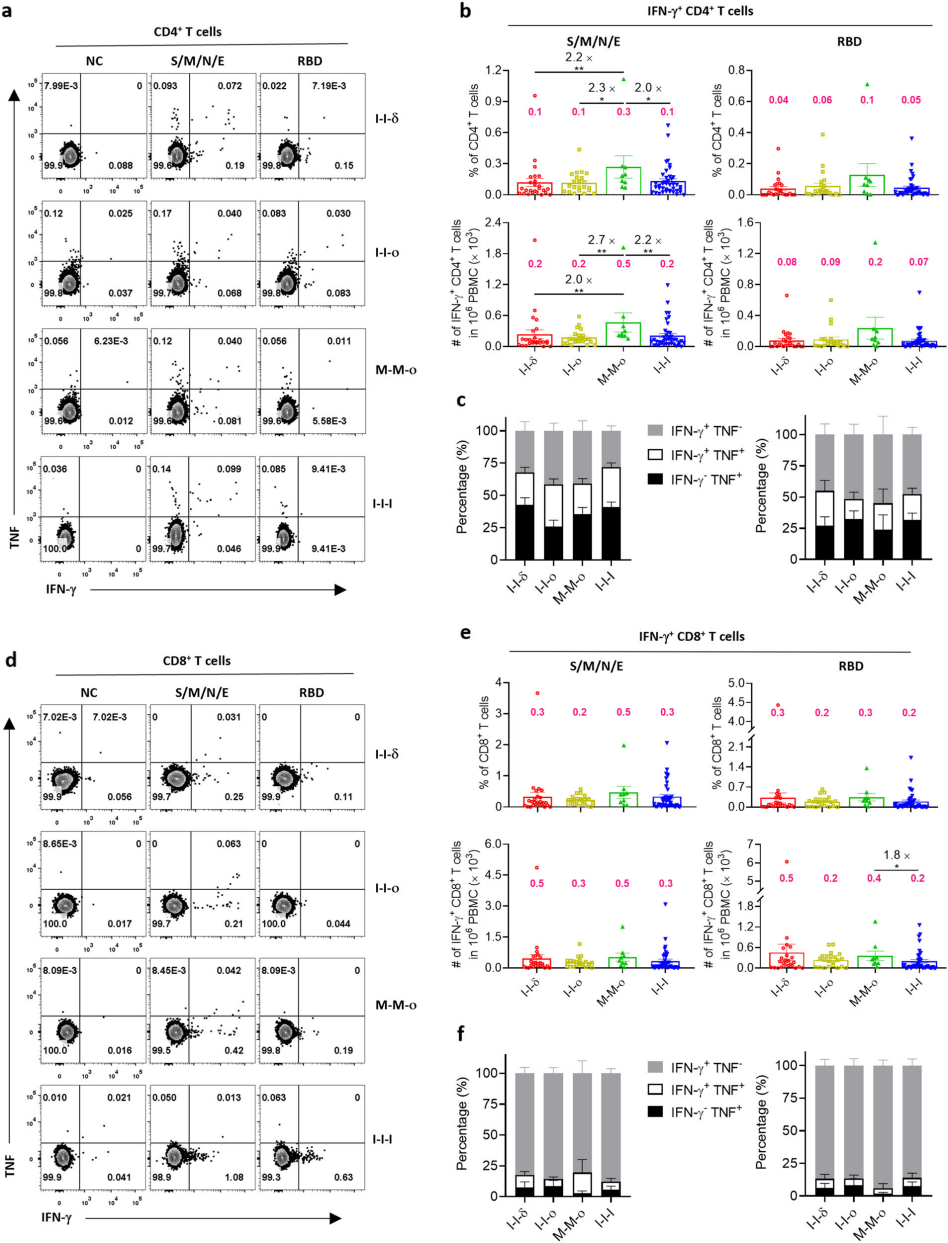
S/M/N/E- or RBD-specific T-cell responses against WT SARS-CoV-2 ex vivo. **a**, **d** Representative flow cytometry plots in the I-I-δ, I-I-ο, M-M-ο, and I-I-I groups showing S/M/N/E- or RBD-specific CD4^+^ T-cell responses (IFN-γ^+^) (**a**) and CD8^+^ T-cell responses (IFN-γ^+^) (**d**) against WT peptide pools of S/M/N/E and RBD. **b**, **e** Frequency and number of all S/M/N/E- or RBD-specific CD4^+^ T cells (**b**) and CD8^+^ T cells (**e**) in the I-I-δ (*n* = 25), I-I-ο (*n* = 21), M-M-ο (*n* = 9), and I-I-I (*n* = 40) groups. The number in magenta indicates the mean in the frequency and number of detected responses. **c**, **f** S/M/N/E- or RBD-specific CD4^+^ T cells (**c**) and CD8^+^ T cells (**f**) expressing IFN-γ and TNF are shown. Comparisons were performed using Mann–Whitney tests. Data in bar charts are shown as the means ± SEM and those in dot plots as the means ± interquartile range. Each dot represents one donor. *P < 0.05, **P < 0.01.

To investigate whether different breakthrough infections and I-I-I vaccination could lead to different levels of SARS-CoV-2-specific T-cell responses, levels of IFN-γ^+^CD4^+^ and IFN-γ^+^CD8^+^ T cells were measured as virus-specific T cells by intracellular cytokine staining after ex vivo stimulation with the peptide pools of S/M/N/E and RBD. The percentage of S/M/N/E-specific (S/M/N/E^+^) CD4^+^ T cells was markedly greater for M-M-ο than I-I-δ (0.3 vs 0.1, 2.2-fold, *P* = 0.0061), I-I-o (0.3 vs 0.1, 2.3-fold, *P* = 0.0463) and I-I-I (0.3 vs 0.1, 2.0-fold, *P* = 0.0364), and the number of S/M/N/E^+^CD4^+^ T cells was also markedly greater for M-M-ο than I-I-δ (0.5 vs 0.2, 2.0-fold, *P* = 0.0061), I-I-o (0.5 vs 0.2, 2.7-fold, *P* = 0.0090) and I-I-I (0.3 vs 0.2, 2.2-fold, *P* = 0.0060) (Fig. 3a, b). Furthermore, only the M-M-ο group showed an increasing trend in the proportion of RBD^+^IFN-γ^+^CD4^+^ T cells (Fig. 3b) and a higher percentage of IFN-γ^+^CD4^+^ T cells among virus-specific CD4^+^ T cells; no notable difference was seen between the groups (Fig. 3c). Unexpectedly, compared to I-I-I, the induced virus-specific CD8^+^ T-cell response was not higher in all breakthrough infections. Indeed, significant differences in the number (0.4 vs 0.2, 1.8-fold) of RBD^+^CD8^+^ T cells were observed only between M-M-ο and I-I-I groups; similar levels of S/M/N/E^+^CD8^+^ and RBD^+^CD8^+^ T cells were induced in different breakthrough infections and vaccinations (Fig. 3d, e). Notably, only a small portion of double-positive (IFN-γ^+^TNF^+^) CD8^+^ T cells were observed in S/M/N/E^+^CD8^+^ and RBD^+^CD8^+^ T cells among all four groups, indicating that the virus-specific CD8^+^ T cells induced by breakthrough infection and three-dose inactivated vaccine lacked multiple functions (Fig. 3f).

### M-M-ο induced more effective proliferation of virus-specific CD4^+^ and CD8^+^ T cells than I-I-ο and I-I-I

**Fig. 4:**
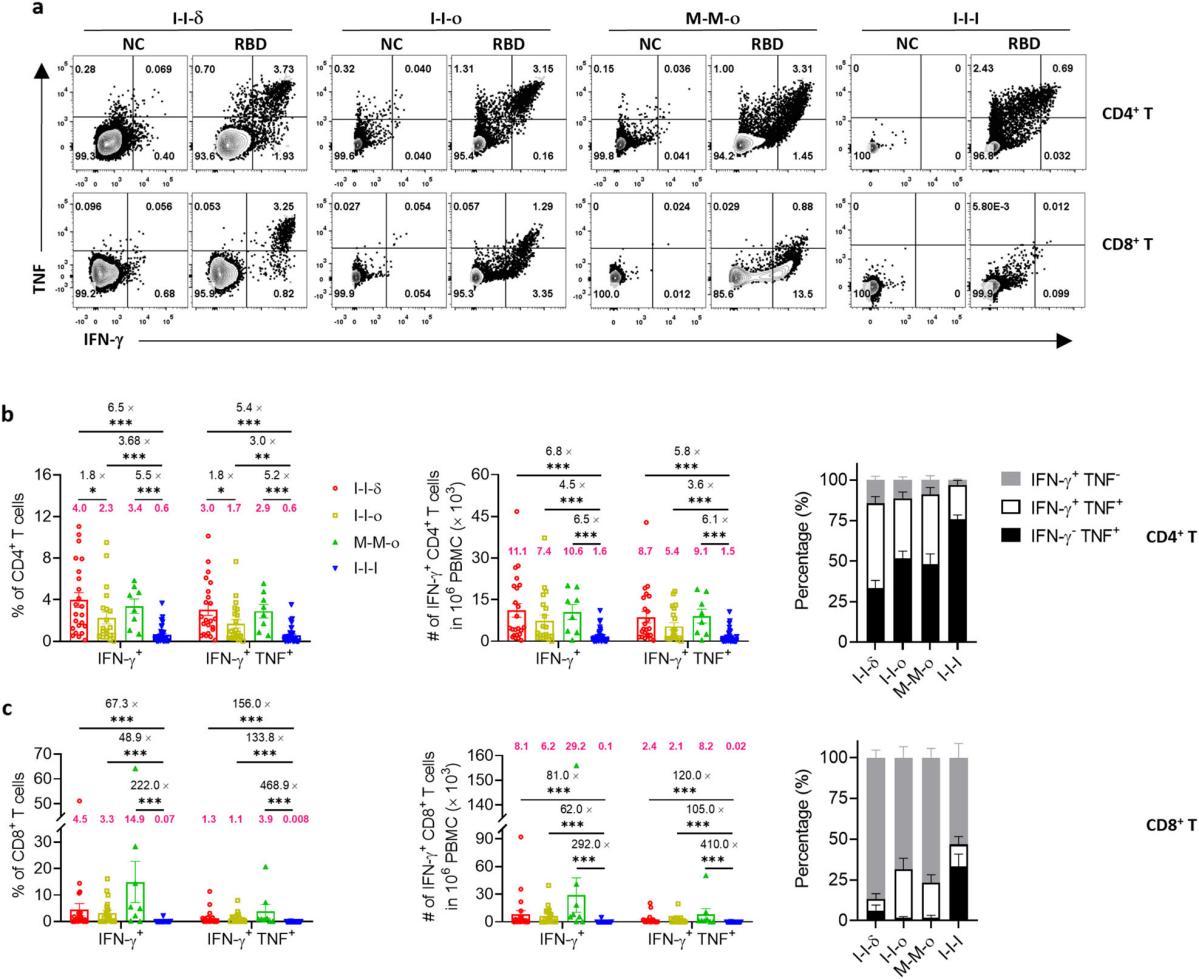
RBD-specific T-cell responses against WT SARS-CoV-2 in vitro. **a** Representative flow cytometry plots in the I-I-δ, I-I-ο, M-M-ο, and I-I-I groups showing RBD-specific CD4^+^ and CD8^+^ T-cell responses against WT RBD peptide pool. **b**, **c** Frequency and numbers of all RBD-specific CD4^+^ and CD8^+^ T cells in the I-I-δ (*n* = 23), I-I-ο (*n* = 21), M-M-ο (*n* = 8), and I-I-I (*n* = 40) groups. The number in magenta indicates the mean in the frequency and numbers of detected responses. Each dot represents one donor. Comparisons were performed using Mann–Whitney tests. *P < 0.05, **P < 0.01, ***P < 0.001.

To explore the proliferative capacity of virus-specific T cells and the robustness of their potential responses to antigens ex vivo, peripheral blood mononuclear cell (PBMC) samples were repeatedly stimulated with pooled peptides in a 10-day in vitro culture, and the numbers and frequency of virus-specific CD4^+^ and CD8^+^ T cells (defined by IFN-γ and TNF) were measured. The RBD^+^CD4^+^ and RBD^+^CD8^+^ T cells proliferated to varying degrees (Fig. 4a–c). Compared with the ex vivo results, RBD-specific CD4^+^ T cells proliferated significantly more in the frequency and numbers for I-I-δ (4.0%, 11.1 × 10^3^/million PBMCs), I-I-ο (2.3%, 7.4 × 10^3^/million PBMCs), M-M-ο (3.4%, 10.6 × 10^3^/million PBMCs), and I-I-I (0.6%, 1.6 × 10^3^/million PBMCs). Among the four groups, RBD^+^IFN-γ^+^CD4^+^ T cells of M-M-ο showed significantly higher proliferation capacity than those of I-I-I in both frequency (5.5-fold) and numbers (6.5-fold). Additionally, the percentage of bifunctional RBD^+^IFN-γ^+^TNF^+^CD4^+^ T cells increased substantially in all groups, whereas monofunctional RBD^+^IFN-γ^+^CD4^+^ T cells were dramatically reduced (Fig. 4b). Concerning the subset of RBD^+^IFN-γ^+^CD8^+^ T cells, the I-I-I group showed the lowest proportion and number of RBD^+^CD8^+^ T cells, regardless of measurement by IFN-γ or by IFN-γ and TNF, compared to I-I-δ (67.3-fold in frequency and 81.0-fold in numbers) and I-I-ο (48.9-fold in frequency and 62.0-fold in number) groups (Fig. 4c). Impressively, RBD^+^CD8^+^ T cells were 222.0-fold higher in proportion and 292.0-fold higher in number in the M-M-ο group than in the I-I-I group (Fig. 4c). The observation that I-I-δ and I-I-o groups showed comparable RBD-specific CD4^+^ and CD8^+^ T-cell responses suggested that variants (Delta or Omicron) did not affect the proliferation capacity of virus-specific T cells (Fig. 4b, c). The abundance of RBD^+^IFN-γ^+^TNF^+^CD4^+^ T cells in the M-M-ο group was higher than that in the I-I-ο group (Fig. 4b), although there was no statistical difference, indicating the superiority of mRNA vaccines over inactivated vaccines.

### Breakthrough infection and vaccination resulted in diverse differentiation patterns of SARS-CoV-2-specific T cells

**Fig. 5:**
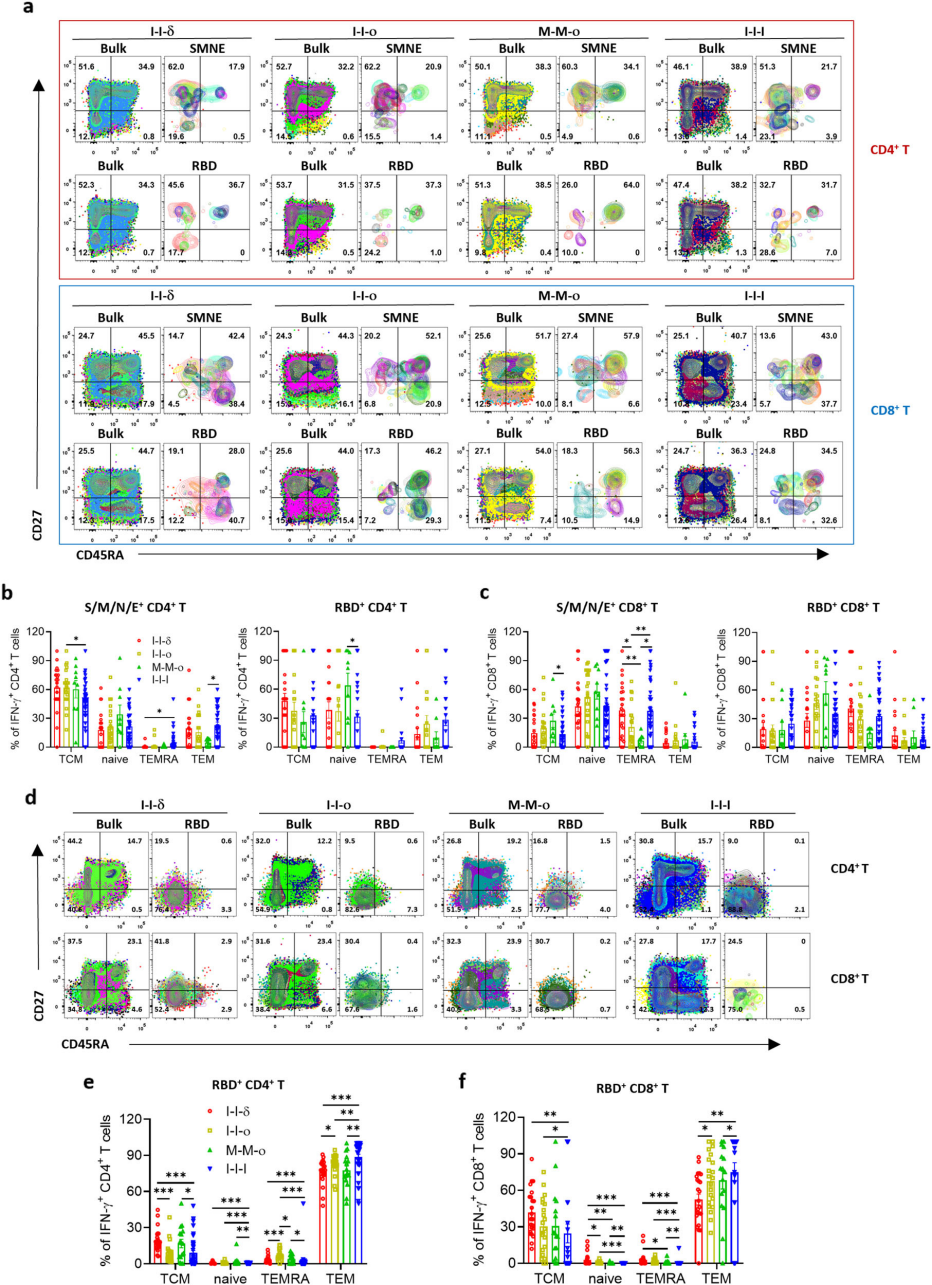
Memory phenotype differentiation of specific memory T cells. **a** Overlay flow cytometry plots in the I-I-δ, I-I-ο, M-M-ο, and I-I-I groups showing S/M/N/E- and RBD-specific memory CD4^+^ and CD8^+^ T-cell phenotypes in ex vivo assay. **b**, **c** Frequency of different RBD-specific CD4^+^ and CD8^+^ memory T cells in the I-I-δ (*n* = 25), I-I-ο (*n* = 21), M-M-ο (*n* = 9), and I-I-I (*n* = 40) groups. **d** Overlay flow cytometry plots showing RBD-specific memory CD4^+^ and CD8^+^ T-cell phenotypes in in vitro assay. **e**, **f** Frequency of different RBD-specific CD4^+^ and CD8^+^ memory T cells in the I-I-δ, I-I-ο, M-M-ο, and I-I-I groups. Data in bar charts are shown as the means ± SEM and those in dot plots as the mean. Each dot represents one donor. Comparisons were performed using Mann–Whitney tests. *P < 0.05, **P < 0.01, ***P < 0.001.

To explore the differentiation patterns of virus-specific memory T-cell subsets among the four groups, two surface markers, CD45RA and CD27, were used to further subdivide memory T-cell subsets into central memory T cells (TCM, CD45RA^–^CD27^+^), effector memory T cells (TEM, CD45RA^–^CD27^–^), CD45RA^+^ effector memory T cells (TEMRA, CD45RA^+^CD27^–^), and naïve T cells (Tnaive, CD45RA^+^CD27^+^). The results showed that TCM and Tnaive were the two main subtypes of S/M/N/E^+^CD4^+^ T cells in all four groups (Fig. 5a, b), with a higher proportion of TEMRA and TEM (*P* < 0.05) and a lower proportion of TCM (*P* < 0.05) in I-I-I than I-I-δ, M-M-o and I-I-o, respectively (Fig. 5b); this suggested that different variant breakthrough infections and vaccinations affected differentiation of virus-specific CD4^+^ T cells differently. Furthermore, the M-M-o group showed the highest numbers for TCM and Tnaive of S/M/N/E^+^CD4^+^ T cells and Tnaive of RBD^+^CD4^+^ T cells among the four groups (Supplementary Fig. S2c), even though no notable proportion difference in cell subtype was found (Fig. 5b). On the other hand, although the I-I-I group showed the highest proportion of TEM and the lowest proportion of Tnaive and TEMRA among 4 groups in in vitro culture (Fig. 5d, e), the numbers of TEM, Tnaive and TEMRA were relatively lower than other groups (Supplementary Fig. S2d).

Regarding virus-specific CD8^+^ T cells, M-M-ο induced the lowest proportion of S/M/N/E^+^CD8^+^ TEMRA and the highest proportion of TCM among the four groups, followed by I-I-ο (Fig. 5c; Supplementary Fig. S2e). In contrast, the S/M/N/E^+^CD8^+^ and RBD^+^CD8^+^ T cells in the I-I-I group were mainly composed of Tnaive and TEMRA cells, which resulted in the lowest proliferation capacity of these cells among the four groups, as measured by 10-day in vitro culture experiments (Fig. 5d, f; Supplementary Fig. S2f).

### The magnitude of virus-specific T cells did not correlate with the level of NAbs against different variants among the four groups

Studies have reported a correlation between SARS-CoV-2-specific cellular and humoral immune responses. It is worth investigating whether a similar correlation exists between individuals who recovered from breakthrough infection and vaccinated individuals after three exposures to antigen, regardless of vaccination or viral infection. Therefore, proportions of IFN-γ^+^CD4/8^+^ T cells, as measured by ex vivo or in vitro stimulation experiments, were analyzed for correlations with NAb levels against WT, Delta, and Omicron variants. Surprisingly, our analysis revealed no correlation between NAb titers and the frequency of virus-specific CD4^+^ T cells or CD8^+^ T cells, either in the ex vivo or in vitro stimulation experiment (Supplementary Figs. S5, S6), suggesting that the potential correlation between cellular and humoral responses may more likely be reflected in Tfh cell-related proteins than in IFN-γ.

## Discussion

After nearly 3 years of the COVID-19 pandemic, the global population has achieved a certain level of sub-population immunity, either by vaccination or infection. In most developed countries, 30%–70% of the population has been infected, and some of these were breakthrough infections. Therefore, people with different infection or vaccination histories, including infection only, vaccination only, vaccination-infection, or infection-vaccination, will have different levels of sub-population immunity in the above sub-populations. As the short duration of vaccine-induced protective immunity and emerging escape variants in the near future will unavoidably cause everyone to be exposed to SARS-CoV-2 infection^26,27^, the host protective immunity of individuals with breakthrough infections after different vaccination strategies and the influence of such “sub-population immunity after breakthrough infections” on emerging immune escape mutants are topics of increasing interest. Here, we described one possibility from the host immune response perspective to explain how the Omicron variant replaced the Delta variant after widespread vaccination. We found that Omicron breakthrough infection led to better immunogenicity in generating higher NAb levels and broader NAbs against WT, Delta and Omicron variants than Delta breakthrough infection (I-I-δ), irrespective of receiving an inactivated (I-I-ο) or mRNA (M-M-ο) vaccine.

In general, the first SARS-CoV-2 spike protein encountered by the host, either through vaccination or infection, shapes the subsequent immune response^28^. Alpha breakthrough infection preceded by two-dose mRNA vaccine resulted in lower protective (neutralizing) antibody responses against the WT strain and Beta variant but induced higher responses against Delta variant when compared to people with WT strain infection after two-dose mRNA vaccine^29,30^. Furthermore, NAb responses against variants decay differentially over time after such mixed spike-antigen encounters. In our study, different vaccination-infection constellations affected both the level and breadth of NAbs and displayed variety in imprinting the magnitude and differentiation of the T-cell response^31^. Most RBD-specific CD8^+^ T cells in the I-I-I group were TEMRA and Tnaive. In contrast, the I-I-δ and I-I-ο groups had a high proportion of Tnaive and TEMRA cells which exhibited a much stronger ability of proliferation and memory phenotype shift (higher proportion of TEM), suggesting that greater inflammation in their surroundings helped T cells quickly differentiate into TEM cells, reflecting a stronger T-cell response in the non-I-I-I groups in vitro. Furthermore, I-I-I induced RBD-specific CD4^+^ T-cell responses comparable to those induced by I-I-δ and I-I-ο but lower than those by M-M-ο. In fact, whole inactivated vaccines contain more viral proteins, such as M/N/E/ORF1, which can theoretically stimulate more virus-specific CD4^+^ T-cell responses than spike- or RBD-targeted vaccines, such as mRNA, subunit protein, and adenovirus vaccines. It seems that the COVID-19 vaccine will be used annually, similar to the flu vaccine, and that the first few vaccinations or infections will be important for imprinting an individual’s host immune response against COVID-19; thus, the use of the inactivated vaccine as the first exposure may not be a poor choice for SARS-CoV-2 immune imprinting. Further, we noticed that more multifunctionality in virus-specific CD4^+^ T cells than CD8^+^ T cells in different breakthrough infections may be due to the immune imprinting from vaccination history, in which I-I could only induce more memory virus-specific CD4^+^ T-cell response rather than memory virus-specific CD8^+^ T-cell response (Supplementary Fig. S2a, b); thus recalled virus-specific CD4^+^ T cells resulted in more multifunctionality than those primary CD8^+^ T cells upon breakthrough infection. In addition, due to the mutation of the Omicron variant RBD different from SARS-CoV-2 WT strain, it may theoretically affect the T-cell response and differentiation. However, we found that there was no difference in cytokine response, memory types and AIM expression between I-I-δ and I-I-o groups which were stimulated with WT or Omicron peptide pools, respectively (Supplementary Figs. S3, S4).

Our data showed that breakthrough infection after different vaccination strategies disturbed the balance of cellular and humoral responses observed in primary SARS-CoV-2 infection but that the IFN-γ^+^CD4^+^ T-cell response correlates with the level of NAbs in MERS-CoV and H7N9 infection. Surprisingly, virus-specific CD4^+^ T cells, as reflected by spike-specific or RBD-specific cells, did not increase from the first to second vaccinations. In particular, we did not observe a robust CD8^+^ T-cell response in any of the four groups; however, a considerable CD8^+^ T-cell response was found in the M-M-ο group, indicative of the T-cell response induced by the current vaccine in use. Hence, future COVID-19 vaccines may need to be strengthened in terms of inducing a T-cell response, especially the CD8^+^ T-cell response, in the first few doses. Meanwhile, due to the limited sample size in each group, no clear imprinting immune patterns were found between groups. Therefore, a study with a big cohort is necessary in future. Overall, our data provided some clues for future vaccine design with respect to the magnitude and breadth of NAbs as well as the T-cell response and differentiation.

## Materials and methods

### Cohort and sample preparation

This study recruited 34 first-breakthrough Delta variant infection convalescent patients (inoculated with 2 doses of an inactivated vaccine, I-I-δ), 22 first-breakthrough Omicron variant infection convalescent patients (inoculated with 2 doses of an inactivated vaccine, I-I-ο), 9 first-breakthrough Omicron infection convalescent patients (inoculated with 2 doses of mRNA vaccine, M-M-ο), and 40 nonhospitalized adult participants (inoculated with 3 doses of inactivated vaccine, I-I-I) (Supplementary Table S1). Blood samples of breakthrough infection convalescents were collected at ~1 month after disease onset or at 14 days after the third dose. This study was approved by the Ethics Commission of Guangzhou First People’s Hospital (No. K-2022-083-02). Written informed consent was obtained from all the enrolled patients. Serum and PBMCs were prepared as previously described^32^.

### Competitive ELISA (cELISA) for detecting NAb clusters

As previously described^5,33^, two NAb clones, 13G2 and 08B3, were harvested from mouse hybridomas, which were prepared from SARS-CoV-2 RBD protein-immunized mice. 13G2 could neutralize the Wuhan-1 strain but not the B.1.351 variant and the epitope were P2B-1G5, P2C-1F11, P2B-2F6 and ACE2, whereas 08B3 could neutralize both strains and the epitope were P2C-1F11 and ACE2 (unpublished data). On the other hand, we used cell-based cELISA (ccELISA) for the detection of NAb clusters. Briefly, A549 human lung carcinoma cells from ATCC were infected with Lenti-GFP-S (6 P) for 48 h, and cells with high GFP expression were sorted by FACS using BD AriaIII and cultured in media supplemented with 0.5 μg/mL puromycin for 15 days. A549 cells stably expressing SARS-CoV-2 were established. The cells were seeded in 96-well plates at 2 × 10^4^ cells/well. After seeding for 24 h, the cells were fixed with 4% paraformaldehyde at room temperature for 20 min, followed by blocking for 2 h with PBST containing 3% BSA at 37 °C. Next, 50 μL of each diluted serum sample was mixed with 50 μL of diluted 13G2-HRP or 08B3-HRP, added to the A549 cells, and incubated at 37 °C for 1 h. After extensive washing, 100 μL of TMB stabilized chromogen (Invitrogen, USA) was added, and the cells were incubated at 37 °C for 10 min, followed by treatment with 50 μL of stopping solution (R&D Systems, USA). Absorbance was measured at 450 nm using a Multiskan GO microplate spectrophotometer (Thermo Fisher Scientific, Waltham, MA, USA).

### SARS-CoV-2 conventional virus neutralization test

A neutralization test for WH-1 (WT SARS-CoV-2) and VOCs, including B.1.617.2 (Delta) and B.1.1.529 (Omicron), was performed in a certified BSL-3 laboratory, as previously described^5^. Fifty microliters of plasma sample were serially diluted, mixed with 50 μL of virus (100 TCID50) in 96-well flat-bottom plates, and incubated for 1 h at 37 °C. VERO E6 cells (1.2 × 10^4^, ATCC, USA) were seeded in these mixtures and incubated at 37 °C for 4 days; the cytopathic effect was examined using a Celigo Imaging Cytometer.

### Pseudovirus-based neutralization assay

SARS-CoV-2 strains were examined and chosen to represent the prototype strain and emerging Omicron variants, including BA.1, BA.2, BA.4/5, with mutations in the spike protein. Neutralization was measured by reduction of the *luc* gene expression, as previously described for the HIV pseudovirus neutralization assay. The 50% inhibitory dilution (NT_50_) was defined as the serum dilution at which the relative light units (RLUs) were reduced by 50% compared to the virus control wells (virus + cells) after subtraction of the background RLUs in the control groups with cells only. Briefly, the pseudovirus was incubated with serial dilutions of the test samples (six dilutions in a 3-fold stepwise manner) in duplicate for 1 h at 37 °C, together with the virus and cell control wells in hexaplicate. Freshly trypsinized cells were added to each well. Following 24 h of incubation at 5% CO_2_ and 37 °C, luminescence was measured, as described in the section for pseudovirus titration. NT_50_ values were calculated with non-linear regression, i.e., log (inhibitor) vs response (four parameters), using GraphPad Prism 8 (GraphPad Software, USA).

### Peptide pool design and preparation

SARS-CoV-2-specific peptides were designed and synthesized as follows. Each peptide was dissolved in DMSO and then pooled, with each at a concentration of 45 μM to form a stock. In total, 487 15-mer SARS-CoV-2 peptides (overlapping by 11 amino acids) spanning the entire antigen region of spike RBD, spike (S), membrane (M), nucleocapsid (N), and envelope (E) proteins were designed using an online peptide generator (Peptide 2.0) and synthesized by GL Biochem Corporation (Shanghai) with a purity > 80%.

### PBMC isolation and ex vivo stimulation

PBMCs were isolated from heparinized whole blood by density gradient sedimentation using Ficoll-Paque according to the manufacturer’s instructions (GE Healthcare, 17-1440-02). The PBMCs (5 × 10^5^) were then cultured in complete RPMI (c-RPMI, RPMI 1640 medium (Gibco) enriched with supplements, including 10% heat-inactivated FBS (Biological Industries, Israel Beit-Haemek), 100 μM MEM nonessential amino acids (Gibco), 100 U/mL penicillin (Gibco), 0.1 mg/mL streptomycin (Gibco), 2 mM L-glutamine (Gibco), 25 mM HEPES (Gibco), 55 μM 2-mercaptoethanol (Gibco) and 1 mM sodium pyruvate (Gibco)). The PBMCs were treated with the peptide pool containing 487 15-mer peptides (250 nM of each peptide) in the presence of 10 U/mL rIL-2 and 1 μM GolgiPlug (BD Biosciences, San Diego, CA, USA) for 16 h at 37 °C with 5% CO_2_. The approach of using a large peptide pool to stimulate PBMCs was based on that developed by Chevalier et al.^34^ and was validated.

### In vitro PBMC expansion, culture, and stimulation

As previously described^32^, 5 × 10^5^ PBMCs were treated with the peptide pool (250 nM of each peptide) and incubated for 10 days for in vitro culture and stimulation. During this culture, half of the medium was replaced with fresh c-RPMI containing 10 U/mL rIL-2 twice per week. The cells were sub-cultured when needed. On day 10, the cells were re-stimulated for 16 h with medium containing the peptide pool (250 nM of each peptide) before being stained for FACS analysis.

### Flow cytometry

In total, 0.5–1 × 10^6^ cells were harvested from the 16-h or 10-day stimulation cultures, washed, and incubated with Live/Dead Aqua V510 for 15 min on ice. The cells were then washed again and surface-stained for 30 min on ice with the following antibodies: anti-CD3-FITC (BioLegend, clone UCHT1, Cat# 561802), anti-CD4-BV421 (BioLegend, clone OKT4, Cat# 317434), anti-CD8-PerCPCy5.5 (BD Pharmingen™, clone RPA-T8, Cat# 560662), anti-CD27-PE (BD Pharmingen™, clone M-T271, Cat# 555441), and anti-CD45RA-APC-H7 (BD Pharmingen™, clone HI100, Cat# 560674). After fixation and permeabilization with Cytofix and Perm (BD Bioscience, Cat# 554714) on ice for 15 min, intracellular staining was performed on ice for 30 min with anti-TNF-PE-Cy7 (BD, clone MAb11, Cat # 557647) and anti-IFNγ-APC (BD Pharmingen™, clone B27, Cat# 554702). After the final wash, the cells were resuspended in 200 μL FACS buffer. A FACSFortessa instrument (BD Bioscience) was used to acquire data, which were analyzed using FlowJo software (Treestar).

### Statistical analyses

All statistical analyses were performed using GraphPad Prism software. Statistical significance was set at *P* < 0.05 (*P < 0.05; **P < 0.01; ***P < 0.001). Student’s *t*-test was used to analyze differences in mean values between groups. The Mann‒Whitney test was employed to compare the central tendencies of two groups (mean or median). Antibody responses are reported as GMTs with the 95% CI. The Wilcoxon rank-sum test was used to compare paired continuous variables not normally distributed. Cutoff values were assigned to evaluate the significance of the *P* value according to the different statistical analysis methods indicated in each figure legend. All values are presented as the means ± SEM.

## Supplementary information

Supplementary information
