# Supplementary material for: SARS-CoV-2 vaccination-infection pattern imprints and diversifies T cell differentiation and neutralizing response against Omicron subvariants
Source: Cell Discov. 2022 Dec 21;8:136. doi: 10.1038/s41421-022-00501-3 (PMC9769462; doi:10.1038/s41421-022-00501-3)
Supplement: Supplementary file 1 — Supplementary information [file 41421_2022_501_MOESM1_ESM.pdf]

Supplementary Table 1: Demographics of 34 I-I-δ patients, 22 I-I-ο patients, 9 M-M-ο patients and 40 I-I-I vaccinators

Category	I-I-δ (n=34)	I-I-ο (n=22)	M-M-ο (n=9)	I-I-I (n=40)
Age	40.2 (37.0-43.5)	40.0 (35.2-44.7)	34.1 (24.3-43.9)	49.2 (45.2-53.1)
Days after disease onset	19.7 (15.8-23.7)	23.2 (19.2-27.1)	22.1 (15.7-28.5)	/
Days after the 3rd vaccine inoculated	/	/	/	14
Gender				
Female	24 (71%)	16 (73%)	5 (56%)	20 (50%)
Male	10 (29%)	6 (27%)	4 (44%)	20 (50%)
Symptom				
Severe ARDS	0	0	0	/
Moderate	2 (6%)	1 (5%)	1 (11%)	/
Asymptomatic	32 (94%)	21 (95%)	8 (89%)	/
Two-dose vaccination time to infection time interval	173.6 (146.1-201.0)	237.8 (185.9-289.7)	180.8 (140.4-221.1)	/
Vaccine	CoronaVac or BBIBP-CorV	CoronaVac or BBIBP-CorV	BNT162b2 or mRNA-1273	CoronaVac or BBIBP-CorV

Supplementary figure 1

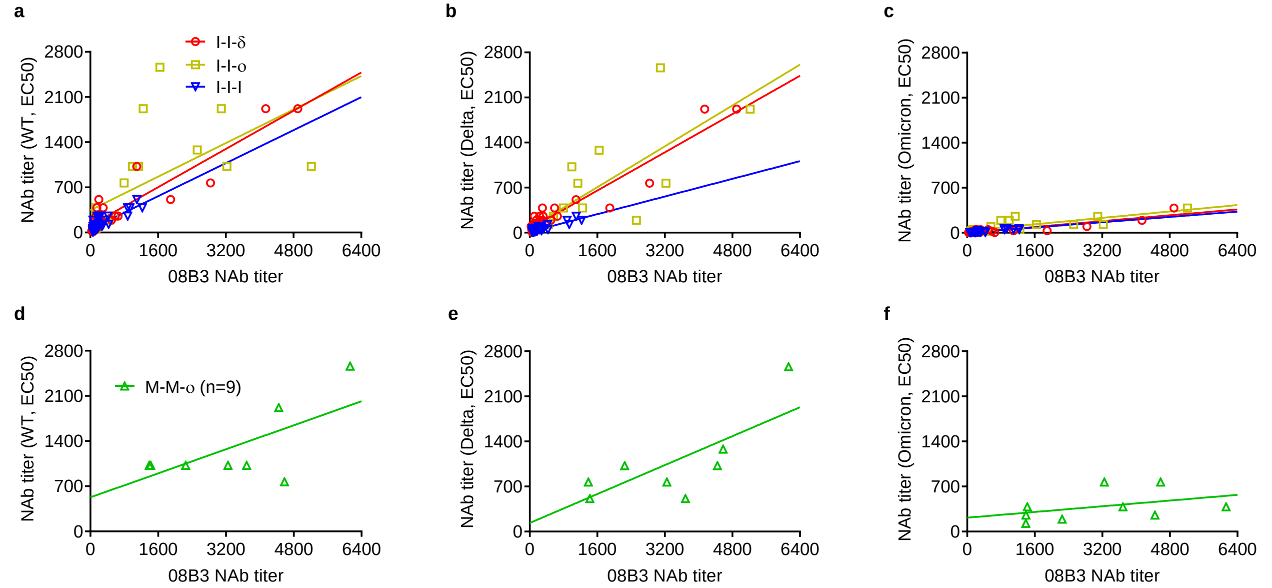

	I-I-δ	I-I-ο	M-M-ο	I-I-I
R^2	0.8620	0.3788	0.4589	0.7530
P	<0.0001	0.0023	0.0450	<0.0001

	I-I-δ	I-I-ο	M-M-ο	I-I-I
R^2	0.9198	0.5658	0.5743	0.8409
P	<0.0001	<0.0001	0.0180	<0.0001

	I-I-δ	I-I-ο	M-M-ο	I-I-I
R^2	0.8243	0.6747	0.1638	0.8576
P	<0.0001	<0.0001	0.28	<0.0001

Fig. S1 | Neutralizing antibody titers against monoclonal antibodies 08B3. a-f The correlation of neutralizing antibodies against the wild-type SARS-CoV-2 (WT) and the Delta and Omicron variants and the monoclonal antibody 08B3 among I-I-δ (n=34), I-I-o (n=22), M-M-o (n=9) and I-I-I (n=40).

Supplementary figure 2

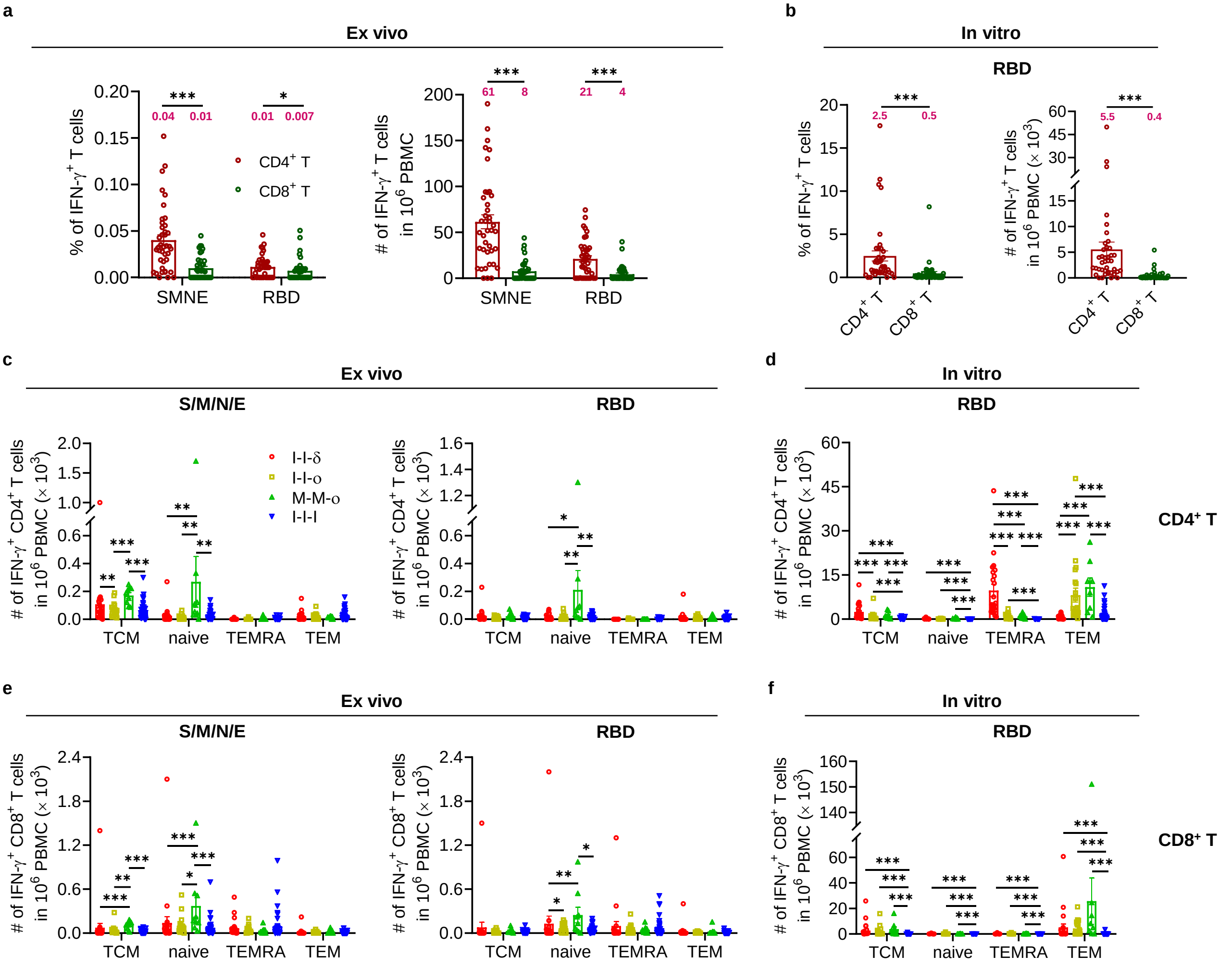

Fig. S2 | The S/M/N/E or RBD-specific T-cell responses (IFN-γ⁺) against wild-type SARS-CoV-2 (WT) peptide pool. a Frequency and number of the SMNE- or RBD-specific T cells from I-I vaccinees (n = 40) stimulated with peptide pools in ex vivo assay. **b** Frequency and number of the RBD-specific T cells from I-I vaccinees (n = 40) stimulated with the WT RBD peptide pool in in vitro assay. **c and e** Comparison of the S/M/N/E or RBD-specific T cell numbers stimulated with peptide pools ex vivo among I-I-δ (n = 25), I-I-o (n = 21), M-M-o (n = 9) and I-I-I (n = 40). **d and f** Comparison of the RBD-specific T cell number stimulated peptide pools in vitro among I-I-δ (n = 23), I-I-o (n = 21), M-M-o (n = 8) and I-I-I (n = 40). The numbers in magenta indicated the mean in the frequency or numbers. Comparisons used Mann Whitney tests. Data in bar charts are shown as mean ± SEM. Each dot represents one donor. *p < 0.05, **p < 0.01, ***p < 0.001.

Supplementary figure 3

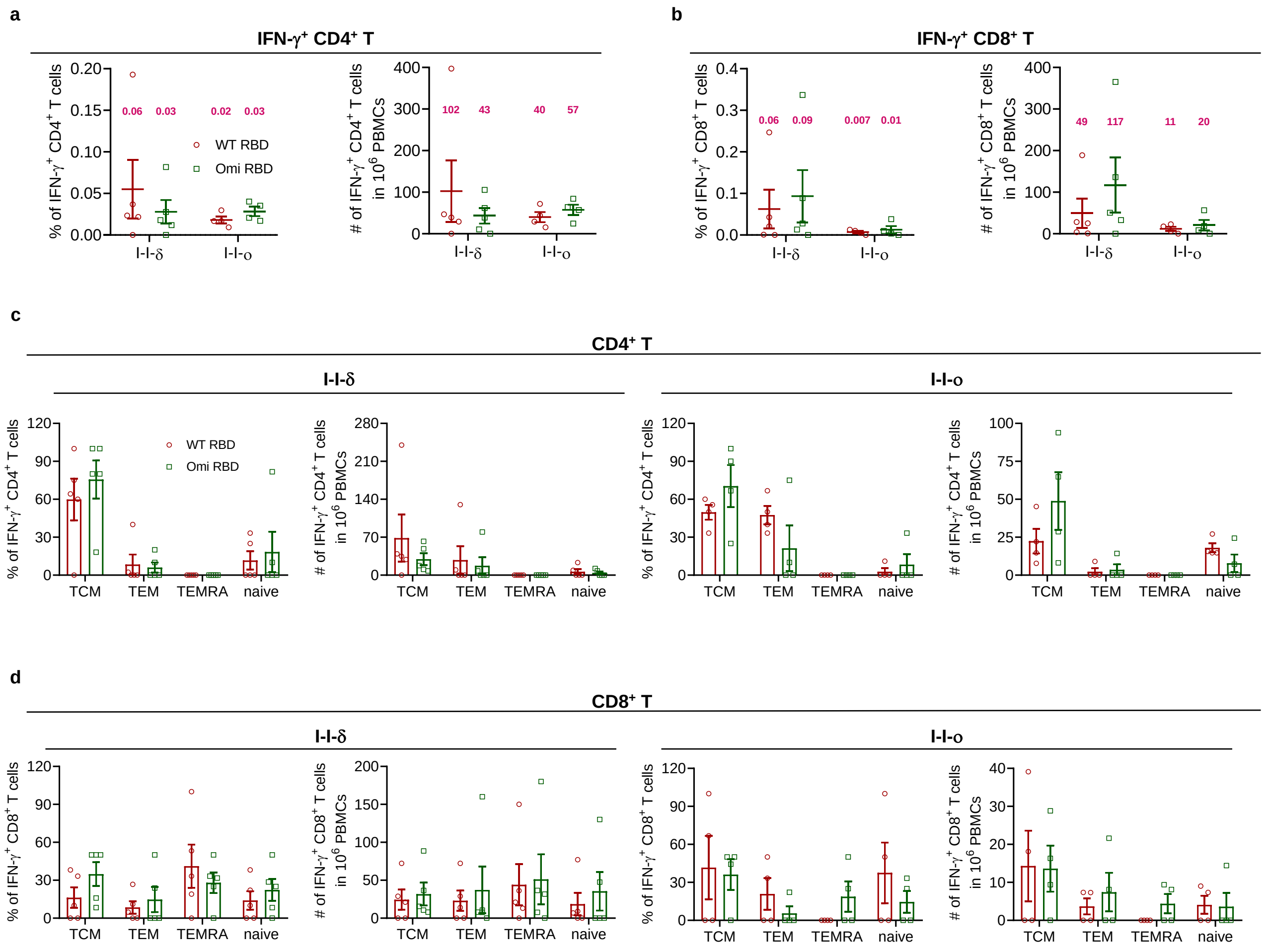

Fig. S3 | The RBD-specific T cell response against wild-type SARS-CoV-2 (WT) and memory T cell phenotype among I-I-δ and I-I-ο. a-b Frequency and number of the RBD-specific **a** $CD4^+$ T cells and **b** $CD8^+$ T cells stimulated with peptide pools of WT RBD or Omicron RBD ex vivo among I-I-δ (n = 5), and I-I-ο (n = 4). **c-d** Frequency and number of different RBD-specific memory **c** $CD4^+$ T cells and **d** $CD8^+$ T cells stimulated with peptide pools of WT RBD or Omicron RBD ex vivo among I-I-δ (n = 5), and I-I-ο (n = 4). The numbers in magenta indicated the mean in the frequency or number. Comparisons used the Wilcoxon rank-sum test. Data in bar charts were shown as mean ± SEM. Each dot represented one donor.

Supplementary figure 4

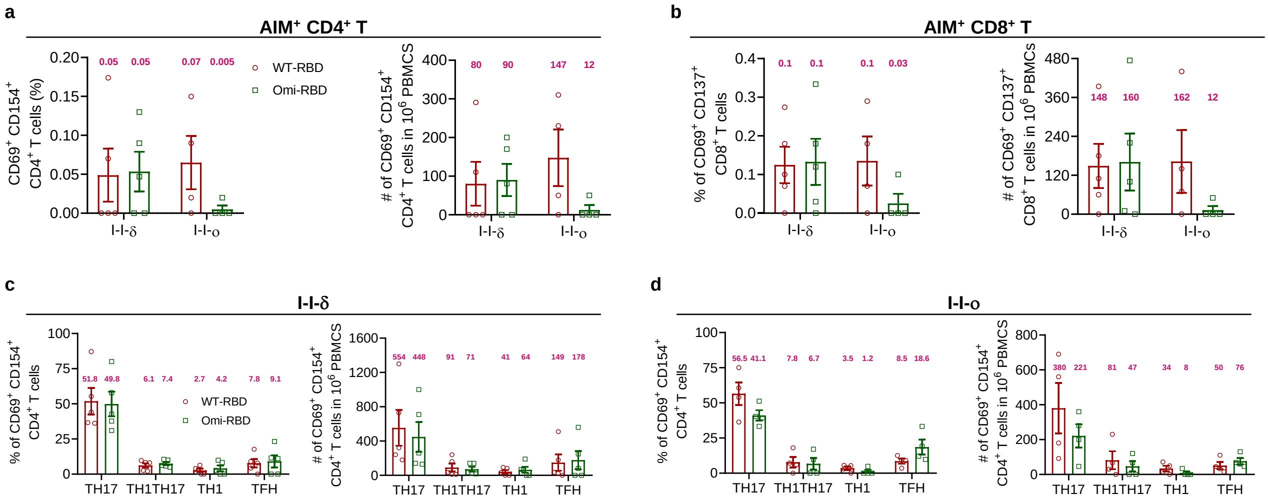

Fig. S4 | The RBD-specific T cell against wild-type SARS-CoV-2 (WT) or Omicron AIM expression and TH differentiation. a-b Frequency and number of the AIM⁺ **a** CD4⁺ T cells and **b** CD8⁺ T cells stimulated with peptide pools of WT RBD or Omicron RBD ex vivo among I-I-δ (n = 5), and I-I-ο (n =4). **c-d** Frequency and number of the RBD-specific TH cell stimulated with peptide pools of WT RBD or Omicron RBD ex vivo among **c** I-I-δ (n = 5), and **d** I-I-ο (n = 4). The numbers in magenta indicated the mean in the frequency or numbers. Comparisons used the Wilcoxon rank-sum test. Data in bar charts were shown as mean ± SEM. Each dot represented one donor.

Supplementary figure 5

I-I-δ	I-I-o	M-M-o	I-I-I

a

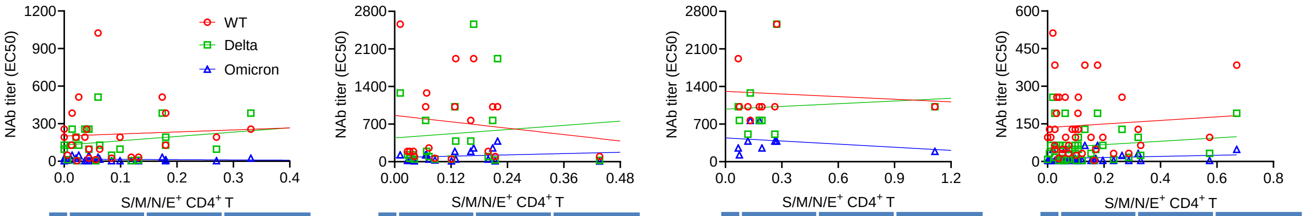

I-I-δ

	WT	Delta	Omicron
R^2	0.003833	0.05083	0.01543
P	0.7738	0.2895	0.5630

I-I-o

	WT	Delta	Omicron
R^2	0.01759	0.008447	0.03685
P	0.5884	0.7082	0.4311

M-M-o

	WT	Delta	Omicron
R^2	0.008329	0.007616	0.0737
P	0.8154	0.8233	0.4798

I-I-I

	WT	Delta	Omicron
R^2	0.006621	0.02311	0.02917
P	0.6177	0.3491	0.2920

b

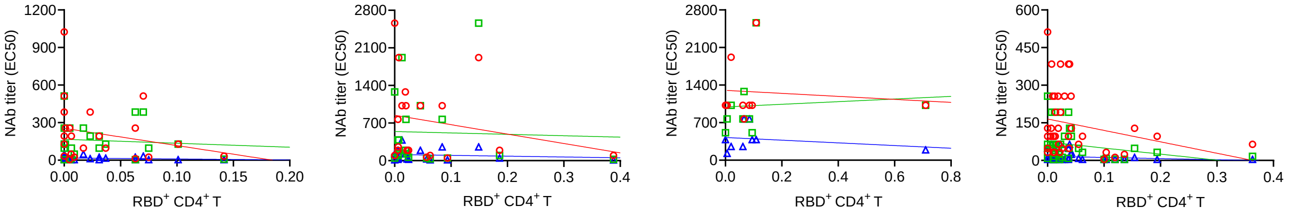

I-I-δ

	WT	Delta	Omicron
R^2	0.05345	0.00812	0.03965
P	0.2771	0.6754	0.3509

I-I-o

	WT	Delta	Omicron
R^2	0.04767	0.001184	0.01812
P	0.3692	0.8888	0.5828

M-M-o

	WT	Delta	Omicron
R^2	0.01146	0.007532	0.05735
P	0.7840	0.8243	0.5349

I-I-I

	WT	Delta	Omicron
R^2	0.06274	0.06825	0.02713
P	0.1190	0.1035	0.3098

c

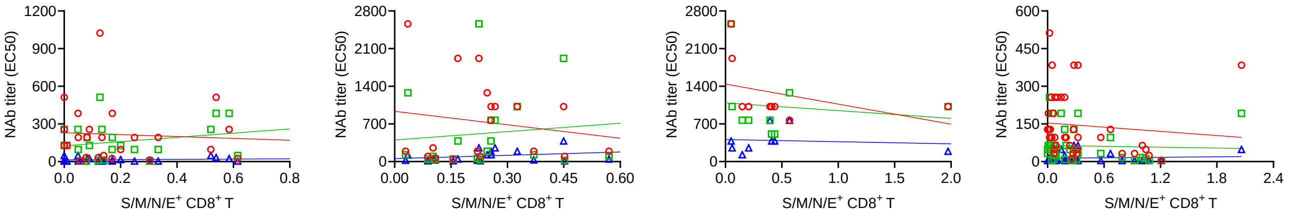

I-I-δ

	WT	Delta	Omicron
R^2	0.003986	0.05835	0.01762
P	0.7695	0.2555	0.5363

I-I-o

	WT	Delta	Omicron
R^2	0.02576	0.01115	0.07948
P	0.5116	0.6670	0.2423

M-M-o

	WT	Delta	Omicron
R^2	0.1437	0.01843	0.01033
P	0.3144	0.7277	0.7947

I-I-I

	WT	Delta	Omicron
R^2	0.009659	0.001643	0.00613
P	0.5463	0.8039	0.6309

d

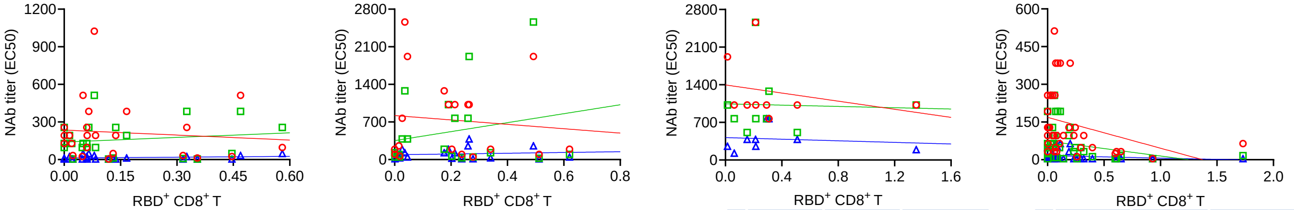

I-I-δ

	WT	Delta	Omicron
R^2	0.009655	0.02438	0.05695
P	0.6478	0.4663	0.2614

I-I-o

	WT	Delta	Omicron
R^2	0.01005	0.04677	0.01516
P	0.6830	0.3738	0.6155

M-M-o

	WT	Delta	Omicron
R^2	0.06889	0.001493	0.01671
P	0.4951	0.9214	0.7403

I-I-I

	WT	Delta	Omicron
R^2	0.1026	0.09273	0.02915
P	0.0439	0.0561	0.2922

Fig. S5 | The correlation of neutralizing antibodies against the WT, Delta and Omicron variants and the T cell response ex vivo. a-d The correlation of neutralizing antibodies against the WT and the Delta and Omicron variants and **a** the S/M/N/E-specific CD4⁺ T cell response, **b** the RBD-specific CD4⁺ T cell response, **c** the S/M/N/E-specific CD8⁺ T cell response, and **d** the RBD-specific CD8⁺ T cell response ex vivo among I-I-δ (n = 24), I-I-o (n = 21), M-M-o (n = 9) and I-I-I (n = 40).

Supplementary figure 6

a

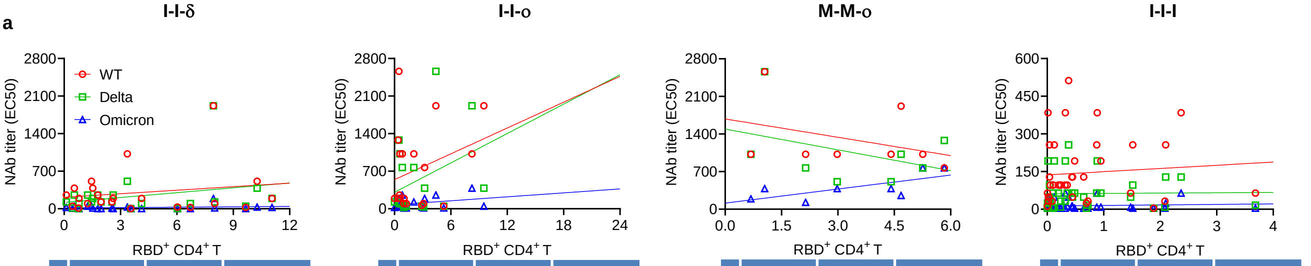

	WT	Delta	Omicron
R^2	0.03169	0.07122	0.04146
P	0.4280	0.2299	0.3634

	WT	Delta	Omicron
R^2	0.08329	0.1205	0.09802
P	0.2308	0.1453	0.1918

	WT	Delta	Omicron
R^2	0.1314	0.1444	0.4974
P	0.3776	0.3532	0.0507

	WT	Delta	Omicron
R^2	0.006937	0.0002873	0.007795
P	0.6094	0.9173	0.5880

b

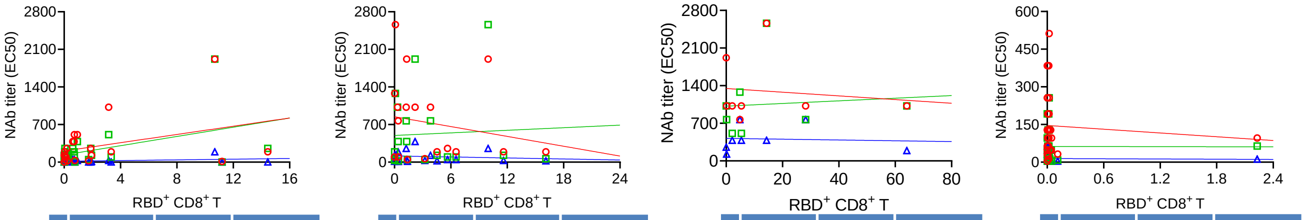

	WT	Delta	Omicron
R^2	0.1345	0.1992	0.1048
P	0.0932	0.0373	0.1417

	WT	Delta	Omicron
R^2	0.03383	0.002604	0.01796
P	0.4510	0.8356	0.5844

	WT	Delta	Omicron
R^2	0.01517	0.00665	0.004556
P	0.7714	0.8478	0.8738

	WT	Delta	Omicron
R^2	0.00491	1.162e-5	0.0007171
P	0.6675	0.9833	0.8697

Fig. S6 | The correlation of neutralizing antibodies against the WT, Delta and Omicron variants and the T cell response in vitro. a-b The correlation of neutralizing antibodies against the WT and the Delta and Omicron variants and the RBD-specific **a** $CD4^+$ T cell response, and **b** $CD8^+$ T cell response; I-I-δ (n = 22), I-I-ο (n = 21), M-M-ο (n = 8) and I-I-I (n = 40).
